# Physician Assistant Students’ Perception of Online Didactic Education: A Cross-Sectional Study

**DOI:** 10.7759/cureus.33833

**Published:** 2023-01-16

**Authors:** Daniel L Anderson, Jeffrey L Alexander

**Affiliations:** 1 Physician Assistant Studies Hybrid Program, Franklin Pierce University, Goodyear, USA; 2 Arizona School of Health Sciences, A.T. (Andrew Taylor) Still University School of Osteopathic Medicine, Mesa, USA

**Keywords:** perceptions of online education, online learning, hybrid medical education, online medical education, physician assistant education

## Abstract

Purpose: This study describes physician assistant students' perception toward online didactic education and highlights relationships between student characteristics and their preference for online learning.

Methods: A previously validated survey questionnaire was administered online to physician assistant students enrolled in traditional, in-person training programs across the United States. The survey consisted of five Likert-scale statements measuring perceptions of online learning and was rated on a seven-point Likert scale. Students also reported their age, gender, history of taking an online course, and preferred learning style. Mean scores were reported for agreement with each Likert-scale statement; Pearson correlation coefficients, one-way ANOVA with post hoc Tukey tests, and independent samples t-tests were used to determine relationships between student characteristics and their preference for online learning.

Results: A total of 391 completed surveys met the inclusion criteria for the study and were used in data analysis. The average age of respondents was 25.98 years, 81.1% (n = 317) were female, 96.2%, (n = 376) reported taking an online course previously, and preferred learning styles were reported as 36.1% (n = 141) visual, 7.7% (n = 30) auditory, 15.6% (n = 61) reading/writing, and 40.7% (n = 159) kinesthetic. Nearly a quarter of respondents indicated they preferred online courses, particularly students with a preferred learning style of reading/writing. No relationships were observed between age, gender, or history of taking an online course and preference for online education.

Conclusion: Most physician assistant students prefer in-person learning. However, a substantial number prefer online learning, and a significant number of these students reported a preferred learning style of reading/writing. More research is necessary to give educational institutions the ability to make data-driven, student-centered program development decisions. However, data in this study indicate a need for continued development of online/hybrid physician assistant programs to better align with current student preferences.

## Introduction

Since the inception of the profession in 1965, physician assistants (PAs) have learned to practice medicine through a combination of in-person didactic instruction followed by the completion of supervised clinical practice experiences at affiliated hospitals and clinics. Although this is an effective curriculum delivery method, pioneers in the field have also recently demonstrated the feasibility of hybrid PA education [1]. In the hybrid model, students complete the didactic year predominantly online, followed by traditional, in-person clinical training. Anderson summarized some of the benefits of online medical education [2]. These include fostering the development of self-directed learners [3], enhancing student engagement in the classroom [4], expanding opportunities for interprofessional education experiences [5,6], promoting digital literacy with medical technology [7], widening the instructor pool [7,8], removing barriers to attending PA school [9], repurposing time spent commuting [4], allowing students to live in and learn about the communities where they may one day practice, and helping to close the gap of clinician shortages in underserved areas [10]. The ability to teach didactic content online may also prove beneficial considering recent changes to the educational landscape following the severe acute respiratory syndrome coronavirus 2 (SARS‑CoV‑2)/coronavirus disease 2019 (COVID-19) pandemic. During this time, remote learning in the didactic year across PA programs increased from 6.4% before the pandemic to 96.8% during the pandemic [11]. During this time, there has also been increasing program director support for online didactic education in PA programs [12]. Now that online didactic PA education is possible, innovative PA educators should seek to determine what method of curriculum delivery students prefer, and analyze these preferences to help guide future program development decisions.

A review of the current literature indicates an overall preference for traditional in-person education over online learning among health professions students, both within and outside of the PA profession; however, research specific to the PA community is limited. In a 2006 seminal study, Day et al. concluded that PA students preferred in-person over online curriculum delivery [13], and in 2009, York et al. observed that 78% of PA students enrolled in a web-based evidence-based medicine course stated they would have preferred in-person lectures instead of, or in addition to, online learning [14]. A review of the literature outside of the PA profession yields similar findings [15-17]. Although video-recorded lectures were found to be of equal or more educational value when compared to in-person instruction, Harvard Medical School students continued to attend traditional lectures if given the choice [15]. Hamilton et al. reported that third-year pharmacy students favored a blended educational approach as opposed to an exclusively online course [16]. A study by Bramer et al. also found that United Kingdom nursing students preferred a balanced mixture of online and face-to-face learning and did not feel that online learning should replace traditional teaching [17].

Further research is needed to explore the current preferences of didactic PA students regarding in-person versus online education to ensure PA program development decisions are student-centered, evidence-based, and data-driven. This is especially important considering the number of new PA programs in development and the number of existing programs that are adapting their curricula to combat current and evolving barriers to medical education. The current study sought to narrow this information gap by determining the curriculum delivery preferences of currently enrolled didactic PA students. Participants were also asked to report their age, gender, history of taking an online course, and preferred learning styles to determine if an association existed between any of these factors and their preference for online education.

## Materials and methods

Study description 

This was a cross-sectional survey study. Risks were minimal and consisted of time lost by students to complete the survey and the potential loss of confidentiality among participants. To mitigate these risks, the survey was designed to be completed in fewer than five minutes, participation was a one-time endeavor with no follow-up, and no personally identifiable information was collected. The primary benefit of the study was insight into the preferences of didactic PA students regarding their preferred curriculum delivery method. This could help current and future programs plan the instructional design of their curriculum. The study proposal was reviewed and approved by the institutional review board (IRB) of A.T. Still University, Mesa, Arizona, United States (approval number: 2021-174), and secondary approval was given by Franklin Pierce University, Rindge, New Hampshire, United States (protocol number: 08252021) for exempt status prior to data collection. 

Study sample

The Physician Assistant Education Association (PAEA) Program Report 35 reported that the maximum capacity enrollment of didactic students in PA programs across the United States was 11,299 [18]. According to a sample size estimator by Qualtrics (Seattle, Washington, and Provo, Utah, United States), 372 individuals from this population needed to be sampled to adequately represent the target population [19]. Inclusion criteria for participation were as follows: students had to be at least 18 years old, be enrolled in a currently accredited PA Program in the United States, and be in their didactic phase of study. Students at the Yale PA online program were excluded from participation as the principal investigator felt they may reasonably have a favorable bias toward online education. The principal investigator currently works for Franklin Pierce University and has previously been employed by the University of South Alabama; therefore, students from these institutions were also excluded to limit participation bias. 

Survey design and distribution

A previously validated survey instrument from O’Malley et al. [20] regarding perceptions of online education was adapted for use in the study, with the author's permission. The adapted survey consisted of two questions to identify eligibility criteria, five Likert-scale questions regarding student perception of online education, and four student characteristic/demographic questions: (i) age, (ii) gender, (iii) history of taking an online course previously, and (iv) preferred VARK (visual, auditory, reading/writing, kinesthetic) learning style [21]. An alphabetical list of currently accredited PA programs by state from the Accreditation Review Commission on Education for the Physician Assistant (ARC-PA) website was used to determine which programs to target for student recruitment [22]. The program director for the first accredited program listed for each state was sent a recruitment email asking whether they would offer participation to their didactic students. Those who agreed were asked to forward a standardized student-specific recruitment email to their currently enrolled didactic students that described the study’s purpose, risks, and benefits, and informed students that participation was voluntary, and completion of the survey was considered their consent to participate. The email also provided a link to the survey should they wish to complete it. If no response was received from a program director after one week, the recruitment email was sent for a second and final time. At the end of every two-week period, the next accredited program listed on the ARC-PA website for each state was contacted. This process continued, every two weeks, until the needed sample size of 372 qualified surveys was completed. 

Data collection, storage, and analysis

Data were collected from September through December 2021. Both ordinal and nominal (categorical) data were collected and stored on the SurveyMonkey platform (Momentive Inc., Waterford, New York). Following data collection, descriptive (frequencies, percentages, means, standard deviations, and confidence intervals) and comparative statistics were conducted using IBM SPSS Statistics for Windows, Version 28.0 (Released 2021, Armonk, New York). To determine if a correlation existed between age and preference for online education, a Pearson correlation coefficient was calculated for each Likert-scale statement; p-values less than .05 were used to determine statistical significance. A one-way ANOVA test was used to compare means among Likert-scale statements according to the four different preferred learning styles of participants; p-values less than .05 were used to determine statistical significance. Post hoc Tukey tests were then conducted to determine pairwise differences between the preferred learning styles for each Likert-scale statement; p-values less than .05 were used to determine statistical significance. Independent samples t-tests were used to determine if there was a significant difference in mean Likert-scale scores between genders or between those who had taken an online course previously versus those who had not; because multiple t-tests were conducted, the criterion for statistical significance was adjusted downward to a p-value less than 0.1 to control for alpha inflation in accordance with the Bonferroni correction.

## Results

PA program participation and student characteristics 

The researchers did not directly access the study sample (didactic PA students), which instead had to be recruited to the study by PA program directors of invited programs. Therefore, the total number of student participants who received the survey was unknown and an accurate response rate could not be calculated. Approximately 35% (50/141) of PA programs invited to the study responded to the email request, and 32% (45/141) opted to participate. These programs represented didactic PA students across 31 states. At the conclusion of data collection, 472 surveys were received, of which 391 were complete, met inclusion criteria for the study, and were subsequently used for data analysis. The average age of the study sample was 25.98 years (range = 21-51 years). Gender was reported as 18.4% (n = 72) male, 81.1% (n = 317) female, 0.3% (n = 1) gender non-binary, and 0.3% (n = 1) preferred not to answer. Most participants (96.2%, n = 376) reported taking an online course previously. The distribution of preferred learning styles of the sample was 36.1% (n = 141) visual, 7.7% (n = 30) auditory, 15.6% (n = 61) reading/writing, and 40.7% (n = 159) kinesthetic (hands-on). 

Participant preferences for online education

Table 1 reports the study participants’ agreement and disagreement with statements pertaining to their perception of online education. Most students disagreed to some degree (strongly disagreed, disagreed, or somewhat disagreed) with four out of the five Likert-scale statements studied; 76.5% (n = 299) disagreed with the statement “Most people believe that online learning is more effective than traditional methodologies”, 72.4% (n = 283) disagreed with the statement “In a course with both traditional and online methodologies, I learn better through the online portion”, 69.4% (n = 271) disagreed with the statement “I prefer online courses to traditional courses”, and 59% (n = 231) disagreed with the statement “I believe that I can learn the same amount in an online course as in a traditional course.” In contrast, most students (56.5%, n = 221) agreed to some degree (strongly agreed, agreed, or somewhat agreed) with the statement “I believe that I can make the same grade in an online course as in a traditional course.”

**Table 1:** Levels of Agreement with Likert-scale Statements Regarding Perception of Online Education n, number of participants

Likert-scale Statement	Strongly Disagree, n (%)	Disagree, n (%)	Somewhat Disagree, n (%)	Neither Agree nor Disagree, n (%)	Somewhat Agree, n (%)	Agree, n (%)	Strongly Agree, n (%)
Most people believe that online learning is more effective than traditional methodologies.	96 (24.6)	125 (32.0)	78 (19.9)	29 (7.4)	39 (10.0)	14 (3.6)	10 (2.6)
In a course with both traditional and online methodologies, I learn better through the online portion.	104 (26.6)	117 (29.9)	62 (15.9)	35 (9.0)	29 (7.4)	27 (6.9)	17 (4.3)
I prefer online courses to traditional courses.	116 (29.7)	96 (24.6)	59 (15.1)	27 (6.9)	41 (10.5)	25 (6.4)	27 (6.9)
I believe that I can learn the same amount in an online course as in a traditional course.	74 (18.9)	83 (21.2)	74 (18.9)	22 (5.6)	55 (14.1)	48 (12.3)	35 (9.0)
I believe that I can make the same grade in an online course as in a traditional course.	41 (10.5)	48 (12.3)	42 (10.7)	39 (10.0)	67 (17.1)	101 (25.8)	53 (13.6)

Relationships between participant characteristics and their preferences for online education 

Age

One survey respondent was omitted from analysis due to an erroneous input of their age (“2t”) which could not be validated. As reported in Table 2, no significant correlation was observed between age and preference for online education. 

**Table 2:** Correlation of Likert-scale Statements with Age n, number of participants

Likert-scale Statement	What is your current age?		
	n	Pearson Correlation	p-value
Most people believe that online learning is more effective than traditional methodologies.	390	0.02	0.66
In a course with both traditional and online methodologies, I learn better through the online format.	390	0.09	0.07
I prefer online courses to traditional courses.	390	0.06	0.23
I believe that I can learn the same amount in an online course as in a traditional course.	390	0.03	0.56
I believe that I can make the same grade in an online course as in a traditional course.	390	0.01	0.79

Gender

Only male and female genders were used in the analysis as these categories made up 99.49% of the sample. As reported in Table 3, no significant relationship existed between gender and preference for online education. 

**Table 3:** T-test Comparison of Likert-scale Scales by Gender n, number of participants; t, independent sample t-test; df, degrees of freedom.

Statement	Gender	n	Mean	Standard Deviation	t	df	Two-sided p-value	Mean Difference	95% Confidence Interval of the Difference		
									Lower Bound	Upper Bound	
Most people believe that online learning is more effective than traditional methodologies.	Male	72	2.64	1.50	-0.16	387	0.87	-0.03	-0.43	0.37	
	Female	317	2.67	1.56							
In a course with both traditional and online methodologies, I learn better through the online format.	Male	72	2.72	1.68	-0.31	387	0.76	-0.07	0.23	-0.52	
	Female	317	2.79	1.75							
I prefer online courses to traditional courses.	Male	72	2.89	1.84	-0.09	387	0.93	-0.02	-0.51	0.47	
	Female	317	2.91	1.92							
I believe that I can learn the same amount in an online course as in a traditional course.	Male	72	3.58	2.07	0.59	387	0.56	0.15	-0.36	0.66	
	Female	317	3.43	1.95							
I believe that I can make the same grade in an online course as in a traditional course.	Male	72	4.33	2.06	-0.44	387	0.66	-0.11	-0.61	0.39	
	Female	317	4.44	1.93							

History of Taking an Online Course Previously

As reported in Table 4, no significant relationship existed between taking an online course previously and preference for online education. 

**Table 4:** T-test Comparison of Likert-scale Statements by History of Taking an Online Course Previously n, number of participants; t, independent sample t-test; df, degrees of freedom.

Statement	Online Course Previously	n	Mean	Standard Deviation	t	df	Two-sided p-value	Mean Difference	95% Confidence Interval of the Difference		
									Lower Bound	Upper Bound	
Most people believe that online learning is more effective than traditional methodologies.	Yes	376	2.66	1.53	-0.74	14.58	0.47	-0.41	-1.59	0.77	
	No	15	3.07	2.12							
In a course with both traditional and online methodologies, I learn better through the online format.	Yes	376	2.77	1.71	-0.79	14.56	0.45	-0.50	-1.85	0.86	
	No	15	3.27	2.43							
I prefer online courses to traditional courses.	Yes	376	2.90	1.88	-0.61	389	0.54	-0.30	-1.29	0.68	
	No	15	3.20	2.31							
I believe that I can learn the same amount in an online course as in a traditional course.	Yes	376	3.44	1.96	-1.86	389	0.06	-0.96	-1.98	0.06	
	No	15	4.40	2.20							
I believe that I can make the same grade in an online course as in a traditional course.	Yes	376	4.38	1.94	-2.74	15.53	0.02	-1.22	-2.17	-0.27	
	No	15	5.60	1.68							

Preferred Learning Style

As reported in Table 5, the highest mean score (most agreement) for each Likert-scale statement was observed with those who selected reading/writing as their preferred learning style; four out of the five differences in means reached statistical significance. There were also statistically significant findings on the pairwise analysis of the different learning styles (Table 6). 

**Table 5:** One-way ANOVA of Likert-scale Statements with Preferred Learning Styles n, number of participants

Likert-scale Statement	Visual	n	Mean	Standard Deviation	95% confidence interval for mean		p-value
					Lower Bound	Upper Bound	
Most people believe that online learning is more effective than traditional methodologies.	Visual	141	2.75	1.49	2.50	3.00	0.04
	Auditory	30	2.70	1.70	2.06	3.34	
	Reading/Writing	61	3.08	1.80	2.62	3.54	
	Kinesthetic	159	2.44	1.44	2.21	2.67	
In a course with both traditional and online methodologies, I learn better through the online format.	Visual	141	2.84	1.71	2.56	3.13	0.02
	Auditory	30	2.77	1.87	2.07	3.46	
	Reading/Writing	61	3.33	2.07	2.80	3.86	
	Kinesthetic	159	2.53	1.56	2.29	2.78	
I prefer online courses to traditional courses.	Visual	141	3.01	1.83	2.71	3.32	0.01
	Auditory	30	2.73	1.91	2.02	3.45	
	Reading/Writing	61	3.56	2.25	2.98	4.13	
	Kinesthetic	159	2.60	1.75	2.32	2.87	
I believe that I can learn the same amount in an online course as in a traditional course.	Visual	141	3.57	1.99	3.24	3.90	0.55
	Auditory	30	3.57	1.96	2.83	4.30	
	Reading/Writing	61	3.66	2.06	3.13	4.18	
	Kinesthetic	159	3.30	1.94	3.00	3.61	
I believe that I can make the same grade in an online course as in a traditional course.	Visual	141	4.43	1.97	4.10	4.75	0.04
	Auditory	30	4.47	2.13	3.67	5.26	
	Reading/Writing	61	5.03	1.75	4.58	5.48	
	Kinesthetic	159	4.19	1.93	3.89	4.49	

**Table 6:** Tukey Post Hoc Pairwise Analysis of Preferred Learning Styles

Likert-scale Statement	Preferred Learning Style (a)	Preferred Learning Style (b)	Mean difference (a-b)	95% confidence interval for mean		p-value
				Lower Bound	Upper Bound	
Most people believe that online learning is more effective than traditional methodologies.	Visual	Auditory	0.05	-0.75	0.85	1.00
		Reading/Writing	-0.33	-0.94	0.28	0.50
		Kinesthetic	0.31	-0.15	0.77	0.30
	Auditory	Visual	-0.05	-0.85	0.75	1.00
		Reading/Writing	-0.38	-1.27	0.50	0.68
		Kinesthetic	0.26	-0.53	1.05	0.83
	Reading/Writing	Visual	0.33	-0.28	0.94	0.50
		Auditory	0.38	-0.50	1.27	0.68
		Kinesthetic	0.64	0.04	1.24	0.03
	Kinesthetic	Visual	-0.31	-0.77	0.15	0.30
		Auditory	-0.26	-1.05	0.53	0.83
		Reading/Writing	-0.64	-1.24	-0.04	0.03
In a course with both traditional and online methodologies, I learn better through the online format.	Visual	Auditory	0.08	-0.82	0.97	1.00
		Reading/Writing	-0.48	-1.17	0.20	0.26
		Kinesthetic	0.31	-0.21	0.82	0.41
	Auditory	Visual	-0.08	-0.97	0.82	1.00
		Reading/Writing	-0.56	-1.55	0.43	0.46
		Kinesthetic	0.23	-0.65	1.12	0.91
	Reading/Writing	Visual	0.48	-0.20	1.17	0.26
		Auditory	0.56	-0.43	1.55	0.46
		Kinesthetic	0.79	0.12	1.46	0.01
	Kinesthetic	Visual	-0.31	-0.82	0.21	0.41
		Auditory	-0.23	-1.12	0.65	0.91
		Reading/Writing	-0.79	-1.46	-0.12	0.01
I prefer online courses to traditional courses.	Visual	Auditory	0.28	-0.69	1.25	0.88
		Reading/Writing	-0.54	-1.28	0.20	0.23
		Kinesthetic	0.42	-0.14	0.98	0.22
	Auditory	Visual	-0.28	-1.25	0.69	0.88
		Reading/Writing	-0.82	-1.90	0.26	0.20
		Kinesthetic	0.16	-0.83	1.10	0.98
	Reading/Writing	Visual	0.54	-0.20	1.28	0.23
		Auditory	0.82	-0.26	1.90	0.20
		Kinesthetic	0.96	0.23	1.69	0.00
	Kinesthetic	Visual	-0.42	-0.98	0.14	0.22
		Auditory	-0.14	-1.10	0.83	0.98
		Reading/Writing	-0.96	-1.69	-0.23	0.00
I believe that I can learn the same amount in an online course as in a traditional course.	Visual	Auditory	0.00	-1.03	1.03	1.00
		Reading/Writing	-0.09	-0.87	0.69	0.99
		Kinesthetic	0.27	-0.33	0.86	0.65
	Auditory	Visual	-0.00	-1.03	1.03	1.00
		Reading/Writing	-0.09	-1.23	1.05	1.00
		Kinesthetic	0.26	-0.75	1.28	0.91
	Reading/Writing	Visual	0.09	-0.69	0.87	0.99
		Auditory	0.09	-1.05	1.23	1.00
		Kinesthetic	0.35	-0.42	1.12	0.64
	Kinesthetic	Visual	-0.27	-0.86	0.33	0.65
		Auditory	-0.26	-1.28	0.75	0.91
		Reading/Writing	-0.35	-1.12	0.42	0.64
I believe that I can make the same grade in an online course as in a traditional course.	Visual	Auditory	-0.04	-1.04	0.96	1.00
		Reading/Writing	-0.61	-1.37	0.16	0.17
		Kinesthetic	0.24	-0.34	0.81	0.72
	Auditory	Visual	0.04	-0.96	1.04	1.00
		Reading/Writing	-0.57	-1.68	0.55	0.56
		Kinesthetic	0.28	-0.72	1.27	0.89
	Reading/Writing	Visual	0.61	-0.16	1.37	0.17
		Auditory	0.57	-0.55	1.68	0.56
		Kinesthetic	0.84	0.09	1.60	0.02
	Kinesthetic	Visual	-0.24	-0.81	0.34	0.72
		Auditory	-0.28	-1.27	0.72	0.89
		Reading/Writing	-0.84	-1.60	-0.09	0.02

## Discussion

Purpose and major findings

The PA educational community has endured many challenges and evolved significantly over the last few years. Due to the unfortunate COVID-19 global pandemic, almost every PA program across the country quickly discovered its ability to implement didactic curriculum online and continue the education of PA students at a time when clinicians were crucially needed [23]. Academic administrators, faculty, students, and other stakeholders must now decide what role hybrid PA programs have in the future of PA education. Perceptions of PA students toward online learning should be considered in this discussion; determining these perceptions was the purpose of this study. 

Most pre-COVID-19 studies show that health professions students generally prefer in-person over online education [13-17], and the results of the current study align with those findings. However, the current study also reveals a significant number of PA students who do prefer learning in an online environment. Despite the majority of participants (69.31%, n = 271) preferring traditional courses, the survey nevertheless identified 23.79% (n = 93) who preferred online learning. Furthermore, as the sample population did not include students from programs that currently use online programming as a major component of their curriculum [2], the preference for online education of the entire PA student population may be slightly higher than reported here. The current study also provides insight into the type of student who may prefer learning online. Interestingly, data analysis showed no correlation between age and students' preference for online education. Similarly, there were no significant relationships between gender or having previously taken an online course and preference for online education. However, a statistically significant relationship did exist between self-reported preferred learning styles and preference for online learning. Although only 15.6% (n = 61) of the sample reported their preferred learning style as reading/writing, this group had the most agreement with all Likert-scale statements favoring online education; four out of five of these results were statistically significant. 

Relevance of findings 

A substantial interest among PA students in online didactic education is not surprising considering other recent studies of medical students in a post-COVID-19 educational environment. Stoehr et al. collected cross-sectional data from 3,286 medical students across 12 countries [24]. In their study, 91% of participants agreed that lecture-style education was a suitable teaching concept for online learning, 97% indicated they had the devices required for online learning, 80% felt comfortable using the software required for online learning, 76% felt well prepared for online learning, and 62% reported being happy with the quality of online courses [24]. Another post-COVID-19 study of 64 medical students at a United States-based allopathic medical school that moved the entire pre-clinical curriculum to a virtual format during the pandemic demonstrated that 70.3% of students reported an unchanged or improved overall medical education in a virtual course module compared to a previous module that was taught in a traditional face-to-face setting [25]. Furthermore, a recent systematic review of 24 studies measuring medical student satisfaction with e-learning during the pandemic indicated that 51.8% of the 15,473 medical students studied were satisfied [26]. 

Study limitations

A potential limitation to this study is that it was conducted during the COVID-19 pandemic and perceptions of online education may have been influenced by either positive or negative experiences encountered during this turbulent time in medical education. Furthermore, the study was not experimental in nature and, therefore, could not control for other variables that may have influenced student perception of online PA education, such as the quality of instructional design and the types of resources made available to students at different institutions. Another limitation was that students were asked to self-report their VARK [21] learning style instead of completing the VARK questionnaire, and students were limited in their selection to only a single preferred learning style. Other limitations to this study are those inherent to survey-based research, including an inability to clarify study questions if needed and not allowing respondents to further explain their answers beyond predetermined survey selections. 

## Conclusions

Most didactic PA students (69.31%) prefer traditional in-person education over online learning. However, the number of didactic PA students that do prefer online learning (23.79%) is also substantial. If we generalize these findings to the 11,299 maximum enrollment slots available per the last PAEA Program Report, there are potentially 2,689 students who may benefit from online/hybrid PA education. Despite these findings, only three out of the 282 currently accredited PA programs are designed to offer a significant portion of their curriculum in an online/hybrid format. Although more research is needed regarding hybrid PA education, based on the findings of this study, the researchers recommend more institutions consider exploring and/or piloting this type of program delivery. Future studies may consider determining the characteristics and preferred learning styles of students who decide to apply to online/hybrid PA programs and how a student’s preferred learning style relates to their satisfaction with, and success in, an online PA educational environment.
